# Effect of free ammonia inhibition on NOB activity in high nitrifying performance of sludge

**DOI:** 10.1039/c8ra06198j

**Published:** 2018-09-13

**Authors:** Fan Zhang, Hong Yang, Jiawei Wang, Ziqi Liu, Qingkun Guan

**Affiliations:** Key Laboratory of Beijing for Water Quality Science and Water Environment Recovery Engineering, Beijing University of Technology Beijing 100124 China yhong@bjut.edu.cn; Beijing General Municipal Engineering Design & Research Institute Co. Ltd. China

## Abstract

The inhibition of free ammonia (FA) on nitrite-oxidizing bacteria (NOB) was investigated using an enriched NOB community with high nitrifying performance. A continuous-flow reactor was operated for the enrichment of the bacterial community. High-throughput sequencing analysis showed that *Nitrospira* (NOB) using in batch experiments was extended from 4.78% to 12.08% during the under continuous-flow operation for 27 days. For each batch experiments, an ammonia injection at the start-up resulted in the desired initial FA concentration (at pH = 8.1–8.2, *T* = 25 °C), and a continuous ammonia feeding stream allowed for a relatively stabilized FA levels as much as the initial one. Results indicated that FA inhibition on NOB was not instantaneous but occurred gradually at a certain reaction time. Low concentrations of FA (18.08–24.95 mg L^−1^) had a limited inhibition on NOB with increasingly high nitrate production rates, whereas high FA levels (36.06–50.66 mg L^−1^) exerted a significant negative impact on the NOB. Also, strong adaptation happened in these high levels of FA inhibition on NOB, which resulted in an overall low NOB activity during the whole aerobic operation.

## Abstract 2

The inhibition of free ammonia (FA) on nitrite-oxidizing bacteria (NOB) was investigated using an enriched NOB community with high nitrifying performance.

## Graphical abstract

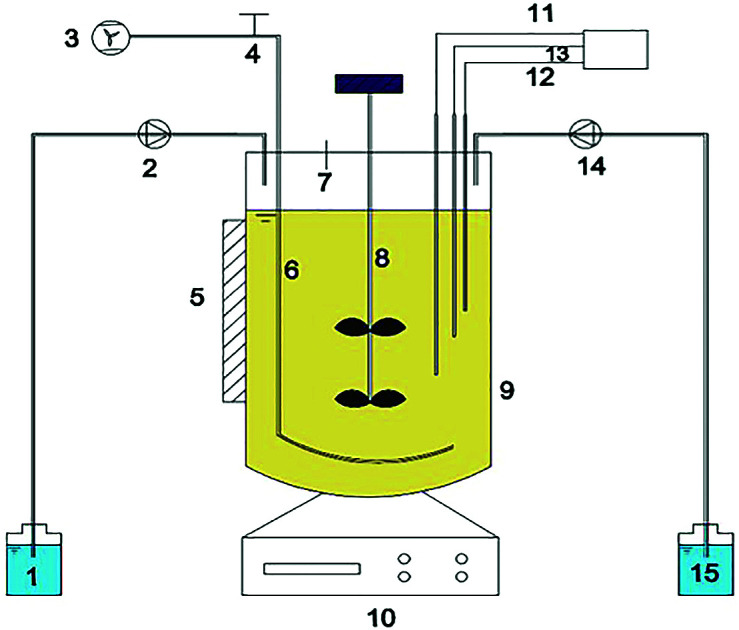

## 1. Introduction

Traditional nitrification is a two-step process commonly utilized in wastewater treatment plant (WWTP). These steps are performed by two different autotrophic bacterial population. First, ammonia is oxidized to nitrite by the ammonia-oxidizing bacteria (AOB) and subsequently, the nitrite is converted to nitrate by nitrite-oxidizing bacteria (NOB). Recently, partial nitrification (ammonia oxidation to nitrite) has drawn much attention as it could reduce the oxygen and organic compound demand by 25% and 40%, respectively, compared with nitrification and denitrification.^1^ The success of the process depends on the elimination of NOB, thereby resulting in nitrite as the primary final product.^2^ Therefore, attempts have been made to devise a microbiological method for AOB enrichment and NOB washout to enable optimal partial nitrification.^3,4^

Some previous works have proved that temperature, pH and dissolved oxygen (DO) are responsible for nitrite build-up.^5–7^ Nevertheless, the direct utilization of temperature (30–35 °C) changes is not feasible in municipal WWTP due to high energy consumption and capital investment. Especially in winter, when the temperature outside is almost close to 0 °C, it is hard to maintain the chamber temperature at 30–35 °C for effective partial nitrification. It has been suggested that high DO concentration improves the ammonia oxidation rate.^8^ Therefore, maintaining a low DO concentration (<0.5 mg L^−1^) for partial nitrification fails to work in highly efficient nitrogen removal systems. Means to maintain high pH and ammonia levels for partial nitrification in WWTP consume huge material resources, thus incurring high operation costs. Therefore, a better solution is proposed using a method for entire NOB washout by bacteria screening from biological system, resulting in the sole existence of AOB.^9,10^ Such sludge used in WWTP treating municipal and high-strength wastewater, achieves saving of operation and infrastructure costs. It is unnecessary to deliberately control low-level of DO, which enables to improve the efficiency of partial nitrification in wastewater treatment.

Washing out the entire NOB population by bacteria screening depends on inhibitors, free ammonia (FA)^11,12^ and free nitrous acid (FNA).^13,14^ Traditionally, FA is the first choice for nitrite build-up and NOB washout. In recent years, some researchers have proposed a new strategy to increase bioenergy recovery from wastewater or sludge treatment using FA.^15–18^ They demonstrate that pre-treatment of the activated sludge with FA for 24–40 h could reduce sludge yields by 20%. At the same time, the NOB activity could be decreased to some extent without any adverse impact on the reactor performance.^19–21^ Consequently, it is imperative that the characteristics and regulations of FA inhibition are discussed.

It is known that the inhibitory effect of FA exerts severe pressure on NOB activity.^22^ Although FA is known to slow down AOB activity, NOB is considered to be much more sensitive to FA than AOB.^23^ Anthonisen *et al.*^13^ suggested that inhibition of NOB begins at a FA concentration of 0.1–1 mg L^−1^, whereas the inhibition threshold for AOB was 10–150 mg L^−1^. FA concentrations of 4 mg L^−1^ and 6 mg L^−1^ have been reported to inhibit the NOB in two different studies.^24,25^ In addition, Abeling *et al.*^26^ reported that NOB was inhibited when FA reached 1–5 mg L^−1^.

The data mentioned above clearly shows that the threshold FA concentration for NOB inhibition varies. However, these FA values were calculated from the initial ammonia concentrations without considering the nitrification rate and ammonia consumption. Hence, the variation in FA concentration is because of the consumption of ammonia under different nitrification rates. Thus, the initial FA concentration should not be considered the real inhibitory value. Very few reports focus on exploring relatively stabilized FA inhibition of NOB with high nitrifying performance in a certain time.

In this paper, a series of batch experiments were designed to establish the relationship between relatively stable FA inhibitory concentrations and NOB response. An enriched NOB culture with high nitrate production rate was used, taken from the continuous-flow reactor with 99% conversion ratio of ammonia to nitrate. The NOB response was assessed systematically by monitoring NOB activity and nitrate production.

## 2. Materials and methods

### 2.1. Continuous-flow operation to enrich NOB

A continuous-flow reactor with a working volume of 95 L was employed to selectively grow NOB for 62 days. Nitrifying sludge from the Gao Bei Dian Wastewater Treatment Plant (WWTP) in Beijing, China was used as the inoculum. This reactor was fed with stock solutions of NH_4_Cl (nitrogen source), KH_2_PO_4_ (phosphorus source) and Na_2_CO_3_ (pH buffer, alkalinity source) as well as a mineral stock solution detailed in Table 1. The 1 mL trace element stock solution contained 0.5 g L^−1^ ZnSO_4_·7H_2_O, 0.5 g L^−1^ MnCl_2_·4H_2_O, 0.4 g L^−1^ CoCl_2_·6H_2_O, 0.4 g L^−1^ CuSO_4_·5H_2_O, 0.2 g L^−1^ NiCl_2_·6H_2_O and 0.05 g L^−1^ Na_2_MoO_4_·2H_2_O. The hydraulic retention time (HRT) was maintained at 5 h. Temperature and pH inside the reactor were set at 25 ± 1 °C and 7.0 ± 0.1, respectively, whereas DO was controlled in the range of 1.0–1.5 mg L^−1^. Throughout the operation period, 99% of the incoming ammonia was oxidized to nitrates. Mixed liquor suspended solids (MLSS) was calculated as 2438 mg L^−1^ on the first day of the continuous-flow culture, which increased up to 4675 mg L^−1^ on day 62.

**Table 1:** Influent characteristic for synthetic wastewater

Compound	Concentration (mg L^−1^)
NH_4_Cl	65–812
KH_2_PO_4_	13–163
Na_2_CO_3_	2000
FeSO_4_·7H_2_O	30
CaCl_2_	15
MgSO_4_·7H_2_O	30
Trace element stock	1 ml L^−1^

### 2.2. Determination of NOB inhibition by FA

#### 2.2.1. Experimental reactor set-up

**Fig. 1:**
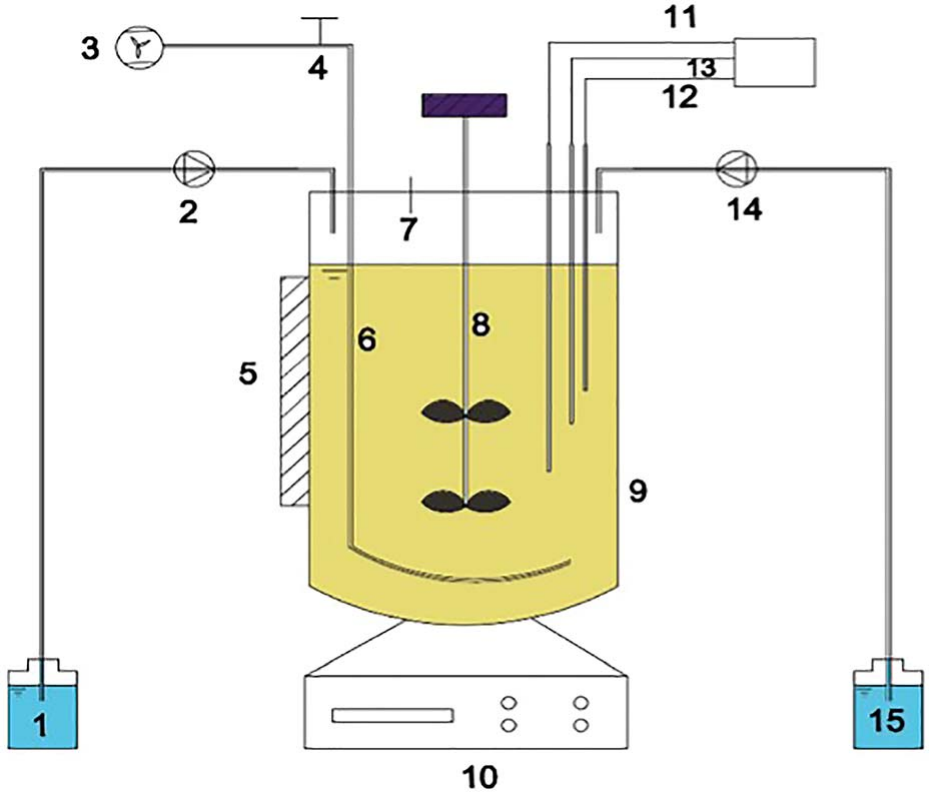
Schematic diagram of the fully automatic miniature fermenter used in this experiment: (1) Na_2_CO_3_ feeding bottle; (2) Na_2_CO_3_ feeding pump; (3) air pump; (4) aeration controlling switch; (5) heat reflecting plate; (6) perforated tube aerator; (7) sampling port; (8) stirrer; (9) main reactor; (10) operation interface; (11) pH probe; (12) temperature probe; (13) DO probe; (14) NH_4_Cl feeding pump; (15) NH_4_Cl feeding bottle.

A fully automatic miniature fermenter (Minifors, INFORS Corp., Switzerland) of working volume 3.5 L as shown in Fig. 1 was used to determine the inhibitory effects of FA. The reactor was fed with NH_4_Cl and Na_2_CO_3_ solutions from two 250 mL feeding bottles using peristaltic pumps that controlled the flow rates. This setup offered continuous streams of ammonia and Na_2_CO_3_, respectively. The pH adjusted automatically at 8.1 ± 0.1 and was controlled by the flow rate of the Na_2_CO_3_ feeding stream. The temperature was controlled at 25 ± 1 °C using a thermostatic heater covering the reactor exterior. Continuous aeration was supplied through a perforated tube using an air pump. The dissolved oxygen (DO) concentration was monitored through a DO probe and maintained at 1.0–1.5 mg L^−1^ by an air flowmeter. The mixture was stirred mechanically at a speed of 200 rpm during the aerobic reaction. The sampling port on top of the reactor allowed for sample collection and feed addition.

#### 2.2.2. Experimental operations

A total of eight batch experiments were performed in a continuous ammonia feeding mode for 3 h each in an aerobic condition in the fermenter. 3.5 L of mixed liquor was removed from the parent continuous-flow reactor in batches across different time points as the nitrate production rate (NPR_0_) reached various levels (Table 2). Ammonia was added in two ways: ammonia was injected into the fermenter at the start-up and a consistent ammonia feed was supplied throughout the operational phase. The specifics of the batch tests are as follows:

**Table 2:** The detail conditions for batch experiments

Test	NPR_0_ (mg (L^−1^ h))	Initial FA (mg L^−1^)	Initial NH_4_^+^–N (mg L^−1^)	MLSS (mg L−1)
1	72.04	18.13	190.32	2867
2	101.57	20.86	244.07	3033
3	115.78	22.13	280.78	3566
4	128.05	24.93	307.61	3896
5	133.65	36.58	451.36	4273
6	142.64	40.75	502.83	4412
7	143.35	44.04	543.41	4415
8	154.06	50.66	625.03	4675

(i) Before each experiment, two sludge samples with same characteristics were collected from the continuous-flow reactor simultaneously. Their MLSS was consistent with that in continuous-flow reactor. All samples were washed thrice with deionized water so that NH_4_^+^–N, NO_2_^−^–N and NO_3_^−^–N were not detected. One sample was injected with a certain concentration of NH_4_^+^–N solution (*e.g.*, 80 mg L^−1^ for Test 1) followed by 1 h operation to attain the actual NPR_0_ (Blank I). The other sample was used to determine the inhibitory effects of FA depending on the actual NPR_0_.

(ii) At the beginning of the test, a certain volume of NH_4_^+^–N stock solution was added into the reactor, resulting in different initial NH_4_^+^–N concentrations: 190.32, 244.07, 280.78, 307.61, 451.36, 502.83, 543.41 and 625.03 mg L^−1^, which were corresponding to the initial FA concentrations: 18.13, 20.86, 22.13, 24.93, 36.58, 40.75, 44.04 and 50.66 mg L^−1^ at pH 8.1 ± 0.1, respectively (Table 2). At the same time, the heater, stirrer and aeration devices were turned on to initiate the reaction.

(iii) Ammonia was fed to the sample during the whole 3 h aerobic reaction. The flow rate was controlled at 83.3 mL h^−1^ in order to ensure nitrification in 3 h. Concentration of the NH_4_^+^–N feed stream was three times the actual NPR measured before the experiments, which were 216.13, 304.71, 347.34, 384.15, 400.95, 427.92, 430.05 and 462.18 mg L^−1^, respectively. The ammonia feed stream could consistently supply NH_4_^+^–N and NO_2_^−^–N to complete the nitrification process. Thus, the NH_4_^+^–N concentrations in the reactor were almost maintained at the same level with the initial NH_4_^+^–N concentrations. The desired FA levels could be stabilized in the original values. For each FA inhibition test, liquor samples were collected at different times (0, 0.5, 1, 1.5, 2, 2.5 and 3 h) to assess the effect of FA on the sludge with different NPR values. During the aerobic reaction, the NO_2_^−^–N concentration in the reactor increased gradually due to FA inhibition. However, the calculated FNA concentration was below 0.008 mg L^−1^ (*T* = 25 ± 1 °C, pH = 8.1 ± 0.1),^14^ which is less than the inhibitory concentration of 0.02 mg L^−1^. Therefore, free nitrous acid (FNA) had negligible influence on the reaction.

(iv) On reaching 3 h, the heater, stirrer, aeration devices, pH controller and NH_4_Cl feeding pump were turned off in each batch reaction. The mixed liquor was discarded from the reactor. As the continuous-flow reaction progressed, the NPR_0_ of the sludge improved gradually. When the NPR_0_ reached a high value, fresh sludge was withdrawn from the continuous-flow reactor to investigate the inhibitory effect of high FA levels.

### 2.3. Analytical methods

The influent and effluent samples were withdrawn from the continuous-flow reactor on a daily basis and analyzed immediately for NH_4_^+^–N, NO_2_^−^–N and NO_3_^−^–N. Mixed liquor suspended solids (MLSS) was also measured inside the continuous-flow reactor on a daily basis. All analyses were performed according to standard methods.^27^ In the batch experiments, NH_4_^+^–N, NO_2_^−^–N and NO_3_^−^–N were measured for the calculation of FA, FNA and nitrate production rate. DO and pH were monitored in the continuous-flow reactor using programmable logic controller, whereas an installed Mettler-Toledo GmbH DO Probe, (Mettler-Toledo, Switzerland) and pH meter (Mettler-Toledo CH-8902, Switzerland) were used in the batch tests, respectively.

In the batch experiments, concentration of FA and FNA were calculated using eqn (1) and (2) proposed by Anthonisen *et al.* (1976)^13^:1
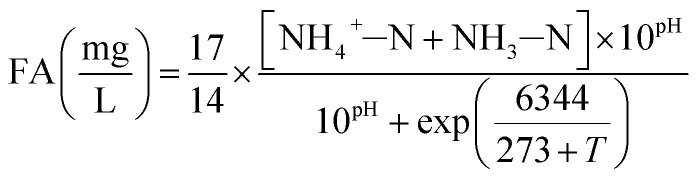
2
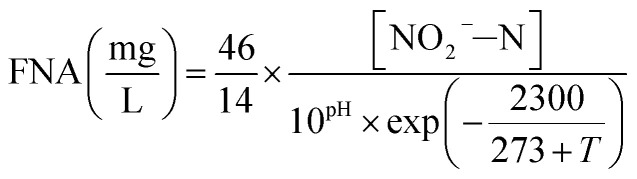
where [NH_4_^+^–N] and [NO_2_^−^–N] are the residual NH_4_^+^–N and NO_2_^−^–N concentrations measured at every 0.5 h interval during the reaction period; thus, FA and FNA concentrations were calculated at 0, 0.5, 1, 1.5, 2, 2.5 and 3 h, respectively.

The nitrate production rate (NPR_0_) in the batch experiments was estimated according to eqn (3)3
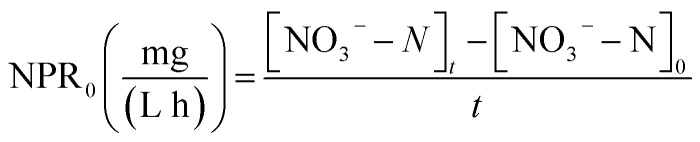
where NPR_0_ is the nitrate production rate measured for Blank I, [NO_3_^−^–N]_t_ is the residual NO_3_^−^–N concentration after reaction time, [NO_3_^−^–N]_0_ is the initial NO_3_^−^–N concentration (the value was zero) and *t* is the reaction time (a constant of 1 h).

FA has a strong negative effect on the biosynthesis process of NOB resulting in the reduction of NOB activity. Hence, the formulae for the calculation of NOB activity in batch experiments were established as follows:4
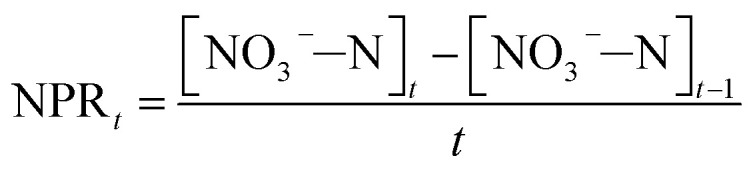
5
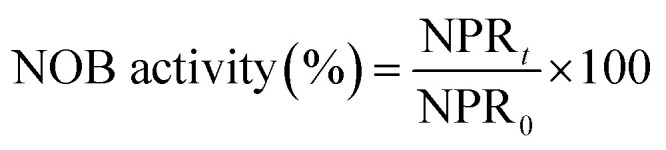
where [NO_3_^−^–N]_*t*_ is the residual NO_3_^−^–N concentration at times 0.5, 1, 1.5, 2, 2.5 and 3 h, [NO_3_^−^–N]_*t*−1_ is the residual NO_3_^−^–N concentration half an hour before [NO_3_^−^–N]_*t*_ was measured (0, 0.5, 1, 1.5, 2 and 2.5 h) and *t* is the 0.5 h reaction time. It should be noted that NOB activity was 100% at 0 h.

### 2.4. High-throughput 16S rRNA gene sequencing

To test the presence of AOB and NOB during the continuous-flow phase, three samples were collected and analysed using high-throughput sequencing (SinoGenoMaxCorp., China). The first sample was taken from the seed sludge, whereas the second and third were withdrawn from the continuous-flow reactor on days 35 and 62. Samples were prepared for DNA extraction. Then, the extracted DNA was supplied for PCR amplification of 16S rRNA in V3–V4 region. The PCR products were verified by electrophoresis to generate sequencing libraries. The libraries were then sequenced and assessed on an Illumina HiSeq2500 PE250 platform. Then, the reads obtained were classified into operational taxonomic units (OTUs), which were subsequently used for microbial diversity analysis.

## 3. Results and discussion

### 3.1. Continuous-flow performance

**Fig. 2:**
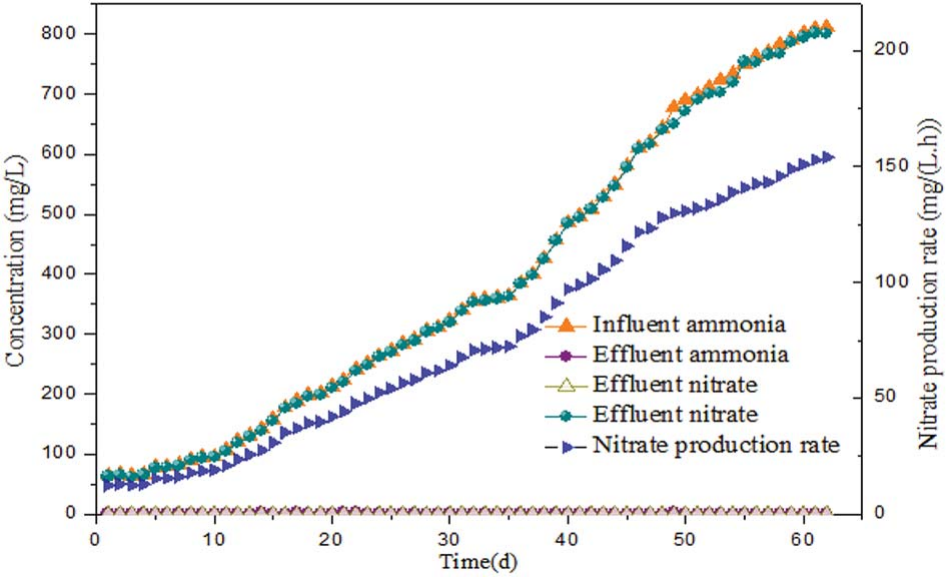
Performance of the continuous-flow reactor.

Long-term monitoring of the reactor performance revealed that complete nitrification was achieved. As illustrated in Fig. 2, influent ammonia was increased in steps from an initial concentration 65 to 812 mg L^−1^. After reaction time of 5 h, the concentration of ammonia was 2.01 mg L^−1^, while that of nitrite was a negligible 0.47 mg L^−1^ in the effluent. Thus, the ammonia was almost completely oxidized into nitrates (over 99%) and the calculated nitrate production rate (the nitrate production rate in this reactor was assessed using the difference of nitrate in the influent and effluent per hour) was only 12.51 mg (L h)^−1^. Low nitrate production rate in the initial phase indicated that the activity of NOB was less and inappropriate for FA inhibition experiments. However, when the influent ammonia was continually improved, it was almost completely transformed into nitrate (99% of ammonia into nitrate transformation ratio during the operation phase), thus reflecting the enhancement of NOB activity. Besides, the output of nitrite was maintained 0.24–0.56 mg L^−1^ through the 62 days. When the nitrate production rate reached 72.04 mg (L h)^−1^ on day 35, the mixed liquor was collected for FA inhibition experiments. With the increase of nitrate production rate in the following days, the sludge was periodically removed for use as the bacterial source for FA inhibition experiments. Additionally, MLSS stepped up from the initial 2438 mg L^−1^ to a final value of 4675 mg L^−1^ implying enrichment of the NOB population. High-throughput sequencing analysis indicated that the microbial culture of the enriched NOB was *Nitrospira*, which increased by approximately 5.6 times from 2.14% (day 1) to 12.08% (day 62). A significant increase of the NOB fraction (4.78%) was detected on day 35, when the first batch experiment was initiated. As the continuous-flow proceeded, the NOB activity and ratio increased progressively to reach 154.06 mg (L h)^−1^ and 12.08%, respectively, when the final batch experiment was conducted on day 62. Thus, the enriched NOB taken from day 35 to 62 triggered a more evident consequence of the NOB response upon FA.

### 3.2. Effect of low FA concentration on NOB activity

**Fig. 3:**
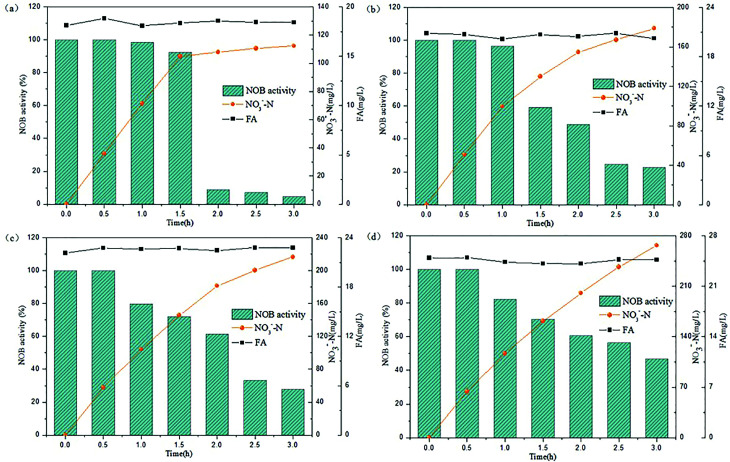
Inhibitory effect of FA on different NPR_0_ values: (a) NPR_0_ = 72.04 mg (L h)^−1^; (b) NPR_0_ = 101.57 mg (L h)^−1^; (c) NPR_0_ = 115.78 mg (L h)^−1^; (d) NPR_0_ = 128.05 mg (L h)^−1^.

Several tests were performed on the samples with varied NPR_0_ under different FA levels (details in Table 2) in order to investigate the biocidal effect of FA on the activated sludge. Fig. 3 reflects the variation of NOB activity in samples with different NPR_0_ values after being exposed to different FA concentrations. FA levels were maintained in a relatively stable range (approximately 18.08–18.83, 20.13–20.86, 22.13–22.81, 24.14–24.93 mg L^−1^) through a continuous mode of ammonia feeding and an ammonia injection at the beginning. The continuous ammonia feeding mode was to ensure the ammonia to nitrate transformation so that the initial ammonia was estimated for FA concentration instead of participating in the transformation of ammonia into nitrate. Therefore, the ammonia concentration was maintained stable during the whole aerobic period resulting in relatively level-off profiles of the calculated FA concentration. The subtle fluctuation in the FA profiles was because the ammonia (assessed for FA levels) was oxidized into nitrate more or less. Since the oxidation of ammonia into nitrate was based on NOB activity, the nitrate production profiles clearly indicated the inhibitory impact of FA on NOB. It should be noted that NOB activity was 100% at 0 h as there was no influence of FA.

As shown in Fig. 3(a), the FA range of 18.08–18.83 mg L^−1^ (Test 1) was selected to investigate the effect on NOB with NPR_0_ of 72.04 mg (L h)^−1^. The NO_3_^−^–N production profile ascended sharply to 104.86 mg L^−1^ upto 1.5 h following by a sudden transition to a slow increase to 112.28 mg L^−1^. Similarly, NOB activity decreased gradually from 100% at 0.5 h to 92.45%, while an unexpected drop happened from 8.85% to 4.61%. This observation correlated well with previous studies,^28–30^ which reported that FA had a negative impact on NOB activity. It could be hypothesized that NOB suppression was not instantaneous but occurred gradually after 1.5 hours. Surprisingly, the percentage of NOB activity decreased intensely reaching 4.61% at the end of the reaction. The results indicated that the FA concentration ranging from 18.08 to 18.83 mg L^−1^ appeared to be the threshold for the sludge at 72.04 mg (L h)^−1^. When the NPR_0_ value reached 101.57 mg (L h)^−1^ (Test 1) as shown in Fig. 3(b), more FA (20.13–20.86 mg L^−1^) was introduced resulting in the rapid improvement of NO_3_^−^–N production before 1 h accompanied by a relatively gradual increase in the subsequent hours. This trend could be due to the fall of FA to the bottom range before 1 h. However, a slight increase of FA level after 1 h allowed for considerable suppression of NOB activity. Therefore, NOB activity was reduced only to 96.48% in 1 h, while an abrupt drop in NOB activity from 96.48% to 22.74% was observed in 1–3 h. In comparison, the NOB activity at NPR_0_ of 115.78 and 128.05 mg (L h)^−1^ corresponding to FA levels of 22.13–22.81 (Test3) and 24.14–24.93 (Test 4) mg L^−1^, respectively, showcased a similar behaviour as well. The NOB activity was not reduced instantly by FA but occurred gradually. Notably, the NO_3_^−^–N production at the end of the aerobic reaction period showed an upward trend from Tests 1 to 4 (112.29, 178.78, 216.79 to 266.74 mg L^−1^). These clearly indicated that the oxidization of nitrite to nitrate was increasingly unrestricted with the increase of NPR_0_, even though FA concentrations were enhanced as well. Moreover, NOB activity also presented the same trend with values 4.61%, 22.74%, 27.87% and 46.83%, respectively, at the end of each test. It could be concluded that FA concentrations up to 24.14–24.93 mg L^−1^ inhibited the NOB population (with high nitrifying performance) to a limited extent. The NOB activity was 100% at 0.5 h in all the above conditions. Subsequently, the NOB activity started to decline sharply in the following hours. It is, therefore, hypothesized that the NOB response for FA inhibition did not occur in a momentary time but in a long term. With the enhancement of NPR_0_, NO_3_^−^–N production increased (corresponding to 112.28, 178.78, 216.79 to 266.74 mg L^−1^ at the end of the four tests) regardless of FA. This clearly implied that low FA concentrations had a limited inhibitory effect on NOB. Furthermore, tests 1 to 4 were vaccinated separately to the mixed sludge from continuous-flow reactor at days 35, 42, 45 and 48, respectively.

### 3.3. Effect of high FA concentration on NOB activity

The above experiments illustrated that the varying trends in NOB activity was observed in a lower FA concentration range below 25 mg L^−1^. Rather, the FA values would increase stepwise in practice because the NPR_0_ values inclined gradually. At this stage, the NPR_0_ was further enhanced to values 133.65, 142.64, 143.35 and 154.06 mg (L h)^−1^, respectively. Thus, NOB inhibition was further investigated at higher NPR_0_ under higher FA concentrations. The inoculum from continuous-flow reactor in Tests 5 to 8 were withdrawn at days 52, 56, 57 and 62, respectively.

**Fig. 4:**
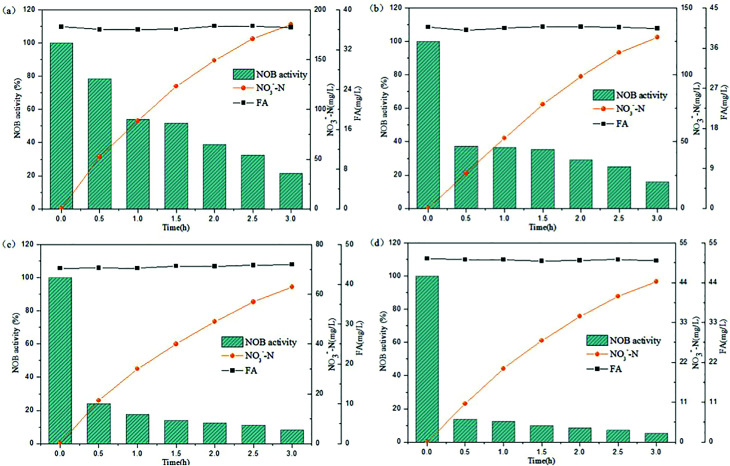
Performance of FA effect on different NPR_0_: (a) NPR_0_ = 133.65 mg (L h)^−1^; (b) NPR_0_ = 142.64 mg (L h)^−1^; (c) NPR_0_ = 143.35 mg (L h)^−1^; (d) NPR_0_ = 154.06 mg (L h)^−1^.

As can be seen in Fig. 4(a), NO_3_^−^–N accumulated gradually reaching a concentration of 185.41 mg L^−1^ when inhibition by FA became apparent in the range of 36.06–36.73 mg L^−1^ (Test 5). Reduction NOB activity to 78.43% occurred at 0.5 h, which was likely due to the strong inhibitory effect of FA at high-levels. In the following hour, the NOB activity began to decline rapidly from 0.5 to 1 h, followed by a slower fall later. Similar result was obtained for the inhibition of NOB by FA at 40.06–40.81 mg L^−1^ concentration, which is depicted in Fig. 4(b) (Test 6). However, NO_3_^−^–N production reached only up to 128.05 mg L^−1^ at the end of the reaction. Furthermore, the NOB activity was estimated as 37.36% at 0.5 h and reduced to 15.93% to a limited extent, which was mainly because of the intense inhibition by FA present at high levels of 40.06–40.81 mg L^−1^. In addition, when the mixed liquor with an initial NPR_0_ of 143.35 mg L^−1^ was collected from the continuous-flow culture on day 57 (Test 7), NO_3_^−^–N accumulation was increased to 63.05 mg L^−1^ in the final reaction phase. Fig. 4(c) demonstrates the NOB performance under a higher FA level of 44.03–44.96 mg L^−1^. NOB activity declined significantly to 24.17% after 0.5 h. Subsequently, a slow descent in the NOB activity led to 8.37%, which represented a suppressed and inactive NOB response due extreme inhibition by such high levels of FA. Interestingly, a further increase of FA concentration to 50.04–50.66 mg L^−1^ (Test 8) did show further inhibitory effect on NOB activity. As shown in Fig. 4(d), the sludge with the highest NPR_0_ of 154.06 mg (L h)^−1^ produced minimal NO_3_^−^–N compared to the other 7 experiments accumulating only 44.35 mg L^−1^ at the end of the reaction. Moreover, the NOB activity dropped suddenly down to 13.76% at 0.5 h and then gradually to 5.31%. According to these results, NOB was inhibited intensely with incremental FA levels in the higher range despite the NPR_0_ increasing only on a small scale. Notably, the high FA concentration at the beginning of the reaction had significant inhibitory effect NOB activity at the startup (0.5 h) in all four experiments discussed above. However, the biomass had started to adapt to the strong inhibition by high in initial stage, thus resulting in the sustenance of NOB activity *i.e.* ammonia to nitrate transformation through the whole aerobic reaction period. This was in agreement with Wong *et al.*,^31^ whose results suggested that NOB adapted to FA above 40 mg L^−1^ and could resist the inhibition.

### 3.4. Microbial diversity analysis

High-throughput sequencing was used to analyze the microbial diversity of the seed sludge and the cultivated sludge for comparison. A coverage 0.96 was achieved in the three samples indicating that the result exclusively represented the microorganisms present in the sludge samples. Abundance estimators Chao1 and ACE were applied to determine the OTUs. Greater richness was reflected by a higher value. Moreover, diversity indices Shannon and Simpson were calculated to understand the diversity of the microbial components. High value for Shannon and low value for Simpson indicated the existence of great diversity in the microbiome. The values of OTUs, Chao1, Shannon and ACE in the seed sludge were 4658, 27 687.51, 8.4 and 69 893.04, respectively (Table 3). A decrease in these values was noted during the 35 days of cultivation showing values 3547, 22 476.63, 4.87 and 52 663.95, respectively. They further decreased to 2773, 18 705.24, 4.21 and 41 317.76, respectively, after 62 days of enrichment. On the contrary, the value of Simpson exhibited an upward trend increasing from 0.02, 0.03 to 0.05. The most feasible explanation for this phenomenon is that the microbial abundance and diversity declined while enriching the sludge for nitrifying bacteria. Meanwhile, nitrifiers had been predominant among the microbial communities in the sample. In other words, the performance of the system and richness of the specifically functional population were improved with a reduction in the microbial diversity as previously reported.^32,33^

**Table 3:** Diversity assessment for each sample^a^

Sample	OTUs	Chao1	Shannon	ACE	Simpson	Coverage
0	4658	27 687.51	8.4	69 893.04	0.02	0.96
1	3547	22 476.63	4.87	52 663.95	0.03	0.96
2	2773	18 705.24	4.21	41 317.76	0.05	0.96

a Sample 0 represented the seed sludge from WWTP; Sample 1 and 2 were cultivated sludge at days 35 and 62.

**Fig. 5:**
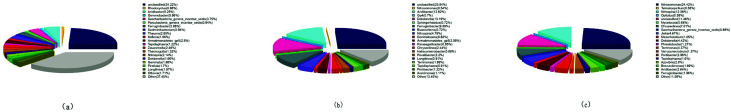
Distributions of microorganisms at the genus level: (a) the seed sludge; (b) cultivated sludge at days 35; (c) cultivated sludge at days 62.

The structure of bacterial community at the genus level is presented in Fig. 5. The seed sludge showed that the *Aridibacter*, *Ferruginibacter* and *Thauera* were the predominant genera as depicted in Fig. 5(a). Their relative abundances were 5.25%, 2.88% and 2.85%, respectively. They belonged to the phyla *Acidobacteria*, *Bacteroidetes* and *Proteobacteria*, respectively, which were the main components of the activated sludge in the wastewater treatment system.^34,35^*Nitrosomonas* (affiliated to β-*Proteobacteria* phylum, called ammonia-oxidizing bacteria) and *Nitrospira* (a distinct phylum, called nitrite-oxidizing bacteria) were detected at 0.43% and 2.14%, respectively, in municipal wastewater treatment.^36,37^ These implied that the abundance of the specific functional species (*Nitrospira*) was very low. Hence, the seed sludge was not suitable for investigating the inhibitory effect of FA. With the increase of continuous-flow operation, the *Nitrospira* improved up to ratios of 4.78% and 12.08%, respectively, at days 35 and 62. Tests 1 to 8 were conducted using increasingly enriched *Nitrospira* from 4.78% to 12.08%. The results were consistent with this showing excellent biological nitrification performance *i.e.* high nitrate production rates ranging from 72.04 mg (L h)^−1^ in Test 1 to 154.06 mg (L h)^−1^ in Test 8. Thus, the negative impact of FA on *Nitrospira* (NOB) is distinct and persuasive. As represented in Fig. 5(b) and (c), the ratio of *Nitrosomonas* (AOB) increased from 0.54% to 24.42% during the operation period of 62 days compared to the seed sludge that contained only 0.43%. The increase in *Nitrosomonas* was 45 times, while *Nitrospira* was enriched 5.6 folds compared to the seed sludge. This is in agreement with the previous reports that NOB grew more slowly than AOB.^38–40^ At the same time, other biological communities were gradually reduced and washed out of the continuous-flow reactor allowing the enrichment of *Nitrosomonas* (AOB) and *Nitrospira* (NOB) communities.

### 3.5. Proposed role of FA inhibition on NOB washout

The compound (FA) had been demonstrated to be the most effective inhibitor for NOB washout.^41^ Previous studies clearly showed that AOB had a much higher level of tolerance to FA compared to NOB, which may have contributed significantly to the elimination of NOB from the biological system. However, considering that wastewater treatment should be economic and environment friendly, it is impossible to employ high levels of FA in municipal WWTP. Thus, FA may provide a feasible solution for the washout of NOB in the lab scale.

Based on the above results, two main aspects about FA inhibition on NOB could be concluded. Firstly, the reaction time of FA inhibition was of supreme importance, as the FA inhibition on NOB was not instantaneous but occurred gradually. As presented in Fig. 3 and 4, the NOB activity declined gradually as the reaction progressed. In addition, lower and higher levels of FA had significantly varied inhibitory effects on NOB. Lower-level of FA (18.13–24.93 mg L^−1^) had a limited inhibitory effect, whereas NOB could develop resistance to some extent towards higher levels of FA (36.58–50.66 mg L^−1^). It has been reported previously that FA concentration up to 200 mg L^−1^ could suppress NOB activity severely.^42,43^ Also, an elevated level of FNA could be supplied for further inhibition of NOB growth resulting in the complete elimination of NOB.^44^ Therefore, combination of high-levels of inhibitors FA and FNA could attain the desired NOB washout. It is imperative to investigate the combined effect of FA and FNA ion the inhibition and NOB washout in subsequent experiments.

It should be mentioned that the NOB culture used in this study coexists with AOB as evident from the high-throughput sequencing, which detected *Nitrosomonas* (AOB) levels of 0.54% and 24.42% at days 35 and 62 compared to 0.43% in the seed sludge. The existence of AOB in the system was inevitable while studying the effect of FA inhibition on NOB. Thus, the response of AOB has not been captured in the data from this study. However, the ammonia fed into the fermenter could transform into nitrate only because of the cooperative activity of AOB and NOB populations. Consequently, the AOB community seems to have still maintained a relative high activity under these FA concentrations. The results (FA inhibition on NOB) obtained may be valid for achieving NOB washout in the presence of AOB. This FA approach could be used to guide AOB enrichment and complete NOB washout in high nitrifying performance systems for efficient and cost-saving partial nitrification in municipal WWTP.

## 4. Conclusions

The feasibility of a continuous-flow configuration was demonstrated for the enrichment of NOB with high performance by conducting a series of experiments of FA inhibition. High-throughput sequencing implied that *Nitrospira* (NOB) used in the batch experiments increased from 4.78% to 12.08% during the continuous-flow operation period corresponding to the high nitrate production rate from 72.04 to 154.06 mg (L h)^−1^. For each batch test, the initial FA generation was through the ammonia injection at the beginning (at pH = 8.1–8.2, *T* = 25 °C) of the culture and the maintenance of these FA levels by a continuous ammonia feeding stream throughout the aerobic reaction period. Results showed that the negative impact of FA on NOB was not immediate but occurred progressively. Low levels of FA (18.08–24.95 mg L^−1^) were found to have limited effect on NOB with increasingly high nitrate production rates. However, much higher levels of FA (36.06–50.66 mg L^−1^) had a strong inhibition on NOB leading to the drastic reduction of activities. Additionally, the NOB was capable of adaption to FA inhibition and thus, the microorganisms were able to remain active at high concentrations of FA. Notably, 18.08–18.83 mg L^−1^ FA concentrations is likely to be the threshold value for the sludge at 72.04 mg (L h)^−1^. It could be suggested that FA inhibitor was effective in NOB suppression and could be applied as an inhibitor for NOB washout where partial nitrification is desired.

## Conflicts of interest

There are no conflicts to declare.

## Supplementary Material
